# Exploring the Emerging Role of Stem Cell Therapy in Neurodegenerative Diseases and Spinal Cord Injury: A Narrative Review

**DOI:** 10.7759/cureus.89629

**Published:** 2025-08-08

**Authors:** Anvvi Kademani, Constantinos Avraam, Daniel Montenegro, Ansu Paloh, Nethra Somannagari, Aarushi Gupta, Ali W Lafi, Alonso E Algaba, Rabeeul Islam, Cheeranthodika Fahima, Humza F Siddiqui

**Affiliations:** 1 Medicine, Balagangadharanatha Swamiji (BGS) Global Institute of Medical Sciences, Bangalore, IND; 2 Neurosurgery, Nicosia General Hospital, Strovolos, CYP; 3 Internal Medicine, Facultad de Medicina Ciencias de la Salud Eugenio Espejo, Universidad Tecnológica Equinoccial (UTE) Equinoctial Technological University, Quito, ECU; 4 Medicine, Tomo Riba Institute of Health and Medical Sciences, Naharlagun, IND; 5 Neurology, Gandhi Medical College, Hyderabad, IND; 6 Medicine, Avalon University School of Medicine, Curacao, CUW; 7 School of Medicine, Washington University of Health and Science, Ramallah, ISR; 8 College of Medicine, Universidad Anáhuac Norte, México City, MEX; 9 Medicine, Calicut Medical College, Kozhikode, IND; 10 Medicine, Southwest Medical University, Sichuan, IND; 11 Internal Medicine, Jinnah Postgraduate Medical Centre, Karachi, PAK

**Keywords:** alzheimer's disease, amyotrophic lateral sclerosis (als), hungtington disease, induced pluripotent stem cells (ipscs), mesenchymal stem cells (mscs), neural stem cells, neurodegenerative diseases, parkinson' s disease, spinal cord injury (sci)

## Abstract

Neurodegenerative diseases and spinal cord injuries (SCI) pose a significant burden on the healthcare system globally. Diseases such as Alzheimer's disease, Parkinson's disease, amyotrophic lateral sclerosis, and Huntington's disease precipitate cognitive, motor, and behavioral deficits. Parallelly, spinal cord injuries produce sensory and motor deficits, which are burdensome psychologically, socially, and economically. Current management strategies focus only on symptomatic relief, with no definitive cure. Stem cells have been explored for regenerative therapy. This review focuses on developments, limitations, and future potential of stem cell therapy. Stem cells affect the central nervous system via neuroprotective mechanisms, immunomodulatory effects, and mitigation of oxidative stress. The clinical implications of stem cell therapy in treating neurodegenerative diseases and SCI are debatable due to varied outcomes. Challenges related to sample size, long-term follow-up, and assessment of adverse effects should be mitigated in future research. Researchers are currently exploring optimal stem cell types along with various transplantation strategies. Biomaterials integrated with stem cells are a novel approach for treating neurodegenerative diseases and spinal cord injuries. Certain genetic modifications have shown improved results. Screening patients to ascertain better responses to therapy has proven to be a challenge. Other complications include graft vs. host reaction and degeneration of transplanted neurons due to pathogenesis and tumorigenesis. However, the majority of the potential stem cell therapeutic avenues are in the preclinical stage and are being tested on animal models. Guidelines pertaining to ethical concerns and regulatory frameworks need to be established to unfold the full potential of stem cell therapy in the clinical setting. Recent advances also show an increased need to formulate patient-specific approaches to treatment, ranging from stem cell selection to the technique of transplantation. Ongoing clinical trials can address the current challenges and leverage emerging technologies, leading to definitive treatments for neurodegenerative diseases and spinal cord injuries.

## Introduction and background

Neurodegenerative diseases and spinal cord injuries (SCI) are major global health conditions that have a great impact on the patients, caregivers, and health systems. Neurodegenerative diseases, including Alzheimer's disease (AD), Parkinson's disease (PD), amyotrophic lateral sclerosis (ALS), and Huntington's disease (HD), are multifactorial chronic irreversible structural and functional diseases of the neuron that advance progressively, causing cognitive, motor, and behavioral deficits [1]. These diseases currently affect millions of people throughout the world. AD accounts for 60-70% of all dementia cases, with over 57 million people throughout the world suffering from dementia [2]. PD affects 8.5 million people globally, constituting about 1% of the population aged over 60 years [3]. Whereas ALS and HD, though much less common, have catastrophic prognoses. The point prevalence of ALS ranges from 1.57 to 11.8 per 100,000 persons worldwide [4]. The estimated global prevalence of HD is 4.8 per 100,000 people [5].

SCI typically occurs due to trauma and may lead to partial or total loss of motor and sensory functions below or at the level of injury [6]. The annual global incidence of SCI is estimated to range from 40 to 80 cases per million, with considerable geographical differences. SCI poses several threats leading to multidimensional consequences besides physical disabilities, including psychological, social, and economic burdens that adversely affect quality of life and increase healthcare costs. Despite advances in medical and rehabilitative care, effective treatments to prevent or reverse neurodegeneration due to the SCI are still lacking [7,8]. Aging populations and increased life expectancy are important contributing factors to the increasing burden of these disorders, highlighting the emerging demand for poignant therapeutic interventions [9]. Current therapies for such neurodegenerative diseases and SCI remain supportive, mainly to relieve symptoms, but do not treat the underlying pathology [6,10]. Lack of definitive treatment strategies significantly contributes to disability-adjusted life years (DALYs) and places an enormous economic burden on patients and healthcare globally [1,9,11]. Investigations of novel therapies, such as stem cell transplantation, gene therapy, and neuroprotective agents, can be promising but require intensive research to demonstrate their safety and effectiveness [11,12].

**Figure 1:**
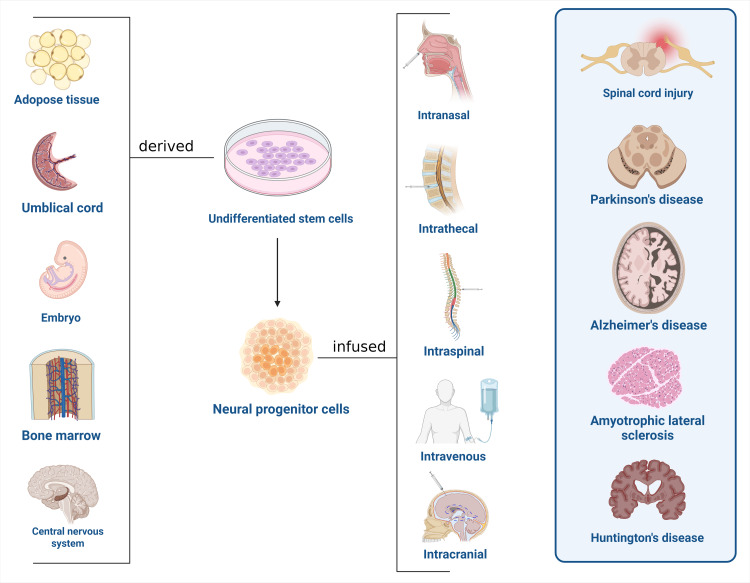
Overview of stem cell therapy in neurodegenerative diseases and spinal cord injury. Figure made by Humza Siddiqui using biorender.com

Stem cells have played a key role in regenerative medicine over the last 50 years, when compared to donated tissues and organs, because of their unlimited ability to divide and transdifferentiate [13,14]. Many types of stem cells are used in regenerative medicine, including embryonic stem cells (ESCs), mesenchymal stem cells, umbilical cord stem cells, bone marrow stem cells, totipotent stem cells, and induced pluripotent stem cells. The mesenchymal stem cells are most preferably used in clinical applications due to their potential for rapid, larger-scale production, easy isolation, and fewer ethical concerns [13,14]. The concept of the application of stem cell exosomes using nanotechnology is gaining popularity in regenerative medicine because of their ability to function in different environments [13,15]. Recently, the Food and Drug Administration (FDA) has approved some stem cell therapies, such as hematopoietic stem cells in leukemia and lymphomas. A serious complication of this transplantation is the steroid-refractory acute graft-versus-host disease, which can be managed with FDA-approved mesenchymal stem cell therapy (SCT) [15]. Conventional treatments for spinal cord injuries and neurodegenerative disorders involving various surgical, pharmacologic, and rehabilitation methods have limited use in correcting the underlying pathology, as they focus mostly on managing the symptoms and slowing disease progression. Stem cell therapies are now widely advocated due to their multiple reparative actions that speed up the regenerative process of the central nervous system [16-18]. (Figure 1). This narrative review aims to summarize the available evidence for the therapeutic potential of stem cells that have been used for neurodegenerative diseases and SCI, outlining the recent developments, limitations, and future potential of this avenue.

## Review

Types of stem cells

A stem cell is a type of cell that has the ability to divide and differentiate into various specialized cell types in the body, including muscles, blood components, and neurons. There are two major types of stem cells in the human body, namely embryonic stem cells and somatic stem cells. The self-renewal ability and multipotency provide the basis of SCT. Stem cells can be derived from different cells and tissues, such as fibroblasts, embryonic stem cells, adipose tissue, and the placenta. They can be cultured in vitro in the presence of certain growth factors in laboratory settings [19]. Various forms of stem cells used in clinical applications include induced pluripotent stem cells (iPSCs), neuronal stem cells (NSCs), neural progenitor cells (NPC), and mesenchymal stem cells (MSCs). The differentiated stem cells from patients themselves can be reprogrammed to generate iPSCs. Subsequently, induced stem cells are transplanted back into the patient, which exhibit anti-amyloidogenic and anti-inflammatory activity and can improve the metabolic activity of neurons [16,20].

Various types of stem cells are found within the body. Pluripotent stem cells have the ability to develop into almost any type of cell in the body. They have the potential to differentiate from all 3 germ layers, including ectoderm, mesoderm, and endoderm. Limbal stem cells are specialized stem cells formed in the limbus, the border of the cornea and sclera in the eye. They are responsible for maintaining and regenerating the corneal epithelium and the outermost layer of the cornea. These stem cells play a vital role in repairing and replacing damaged epithelial cells [21]. Endothelial progenitor cells have the ability to regenerate and repair the endothelium. They reside in the bone marrow and within the blood vessels. These stem cells repair and regenerate blood vessels damaged due to atherosclerosis, aiming to curtail coronary artery disease and stroke [22]. Placental stem cells comprise 2 types, namely mesenchymal stem cells and trophoblast stem cells. Mesenchymal stem cells (MSCs) are found in the amniotic membrane and umbilical cord. Trophoblast stem cells are involved in the formation of the placenta. They play a crucial role in fetal development and maternal-fetal interactions [23]. Neural stem cells (NSCs) reside in specific regions of the central nervous system. They differentiate into nerve cells, astrocytes, and oligodendrocytes. In the adult brain, they are found in the subventricular zone along the lateral ventricle and the subgranular zone of the hippocampus. In adults, they are potentially involved in brain plasticity, learning, memory formation, and repairing damage from injurious insults, such as in the neurodegenerative diseases [24-26].

MSCs have the ability to differentiate into various cell types, including osteocytes, chondrocytes, and adipocytes. Due to their regenerating capability, the placental MSCs are being studied for their potential in treating diseases such as osteoarthritis, cardiac diseases, and autoimmune diseases. Embryonic stem cells are pluripotent cells derived from early stages of an embryo, specifically from a structure called a blastocyst. It has the ability to differentiate into ectoderm, mesoderm, and endoderm. It is used for treating spinal cord injuries, heart diseases, and neurodegenerative diseases. Amniotic MSCs exhibit anti-inflammatory effects and can modulate immune response, which is beneficial in reducing tissue damage and promoting healing. They are being studied for use in tissue engineering, wound healing, and treating various inflammatory and degenerative conditions. Human umbilical cord MSCs are multipotent stem cells isolated from the umbilical cord, typically from a component called Wharton’s jelly. They exhibit low immunogenicity and have the capacity to modulate immune response; hence, they are used for the treatment of autoimmune diseases and inflammatory conditions. These cells have the ability to differentiate and secrete growth factors supporting tissue repair and regeneration [23,27,28]. Bone marrow-derived MSCs can be potentially utilized in bone and cartilage repair and regenerative medicine due to their multipotent self-renewal capacity. Adipose tissue-derived MSCs are extracted from the fat cells. These cells secrete bioactive molecules that can decrease inflammation and modulate immune response, which may contribute to improved healing and reduced tissue damage [29]. MSCs procured from the menstrual blood are an avenue that has been explored due to the non-invasive manner of collection [30].

Stem cell therapies in neurodegenerative diseases

NSCs offer significant potential for regeneration due to their ability to differentiate into various neural cell types. Stem cells, upon direct transplantation into damaged neural tissue, are capable of replacing lost neurons, astrocytes, and oligodendrocytes, potentially restoring lost function and improving neurological outcomes [31]. The newly formed neurons can integrate into existing neural circuits by establishing synaptic connections, facilitating functional recovery. Beyond cell replacement, stem cells contribute to neuroregeneration through neuroprotective mechanisms such as secreting neurotrophic factors, which promote neuronal survival and resilience to injury. They also mitigate oxidative stress by producing antioxidants and modulating protective cellular pathways [32]. Stem cells offer immunomodulatory effects by influencing microglia, reducing inflammation, and promoting a neuroprotective environment [33]. Furthermore, stem cells support neuroregeneration through paracrine effects, releasing neurotrophic factors such as brain-derived neurotrophic factor (BDNF), nerve growth factor (NGF), and glial cell-derived neurotrophic factor (GDNF) that support neuronal survival, growth, and differentiation [34]. Stem cells enhance axonal growth and synaptic plasticity, essential for restoring connectivity and function, by promoting axonal regeneration and contributing to the strengthening or weakening of synapses over time [35].

Alzheimer's Disease

In the realm of neurodegenerative diseases, the prime target is Alzheimer’s disease (AD). The reduction of Aβ42 deposits, enhanced neuronal survival, and the improvement of cognitive functions such as memory and learning abilities have been beneficial due to the usage of the extracellular vesicles derived primarily from bone marrow mesenchymal stromal cells (BM-MSC-EVs) and human adipose-derived stromal/stem cells (hASCs). NSC transplantation targets primarily the neuronal networks and pathological proteins in AD [32,33]. Different stem cell populations can trigger anti-inflammatory signals that slow the progression of Alzheimer’s disease. Stem cells can also be genetically modified to simultaneously address both amyloid-beta plaques and tau tangles or to enhance their neurotrophic and neuroprotective properties. NSCs offer a practical method for delivering therapeutic Aβ-targeting proteins to the brain. A 2015 study by Ager et al. demonstrated that transplanting human central nervous system stem cells (hCNS-SCs) into 3xTg mice resulted in the differentiation of these cells into neural stem cells, immature neurons, and glial cells, along with increased synapse density. While memory consolidation improved, as shown by the Morris-Water maze and novel object recognition tests, the levels of amyloid-beta and tau protein remained unchanged. These findings suggest that differentiation into neuronal cell lines alone does not significantly contribute to cognitive recovery and that hCNS-SC transplantation primarily addresses symptom reversal [36]. The beneficial effects of NSCs appear to stem more from increased synaptic density, restoration of local neuronal numbers, and/or increased neurotrophic factors rather than a reduction in pathological protein levels [37]. Therefore, the long-term efficacy of this approach remains a question. Further research is needed to understand the potential role of NSCs in aggregating damaged proteins, perhaps through glial cell mediation, inflammation, and synaptic rescue. Despite these challenges, NSCs may be crucial for advancing Alzheimer's disease treatment [38].

Chronic inflammation has been shown to play a crucial role in Alzheimer's. The MSC has the ability to express anti-inflammatory factors such as interleukin-10 and prostaglandin. In vitro, human MSCs had a significant enhancement in the number of hippocampal neurons. On the other hand, MSCs have the capability to diminish the amount of alpha-beta 42 by the promotion of autophagy. Using a β-amyloid-induced rat model, researchers investigated the potential of human umbilical cord mesenchymal stem cells (hUMSCs) and mesenchymal adipose tissue stem cells (hAD-MSCs) to improve neurogenesis and synaptic function. The transplantation of MSCs led to a decrease in β-amyloid deposits in the hippocampus of the AD rats. Furthermore, transplantation of both hUMSCs and hAD-SCs significantly reduced the apoptosis rate of hippocampal neurons. This can be safely achieved via intravenous injections [36,37]. Generating human iPSCs has been a technique established in the new era of neurodegenerative disease research. The usage of iPSCs as an alternative to the use of embryonic stem cells avoids many ethical considerations. The CRISPR/Cas9 system has been revolutionary in editing eukaryotic genomes and introducing disease-related mutations into iPSC lines. iPSCs in the Alzheimer's disease model mimicked in succession the pathological state and were able to be utilized to investigate novel treatments, such as combining bromocriptine, cromolyn, and topiramate as an anti-alpha beta cocktail. This anti-alpha-beta cocktail has been shown to reduce the alpha-beta levels by more than 60%. Similar findings are achieved by secretase inhibitor treatment [38].

The phase I clinical trial enrolled nine patients to evaluate the dose toxicity of stereotactic injection of human umbilical cord-derived stem cells (NCT01297218 and NCT01696591). At the 12-month follow-up, the most prevalent complaints were wound pain and headache. No notable adverse events were reported [39]. Nine individuals with mild to moderate Alzheimer's disease dementia participated in the clinical trial (NCT02054208). The Ommaya reservoir was placed into the patient's right lateral ventricle four weeks prior to the administration of MSC. Six patients received high-dose human umbilical cord blood-derived mesenchymal stem cells (hUCB-MSCs), while three patients received low-dose. Three additional MSC injections were given to each of the nine patients, separated by four weeks. These patients were monitored for 36 months. The findings demonstrated that it was adequately safe and well tolerated to triple-inject hUCB-MSCs into the lateral ventricle through the Ommaya reservoir [40]. The Phase 2a clinical trial was conducted to evaluate the efficacy and safety of bone marrow-derived allogeneic MSCs in AD with single and 3-dose infusions of 25 million cells and 100 million cells (NCT05233774). No transplant-related hypersensitivity and deleterious effects of SCT were reported. At 39 weeks post-infusion, the patient showed a reduction in neuroinflammation and deceleration in the decline of brain volume. Patients manifested improvements on the Montreal Cognitive Assessment and the Alzheimer's Disease Cooperative Study Activities of Daily Living [41]. Mild AD Patients were infused with low- and high-dose MSCs in a phase I clinical trial. Participants in the low-dose group showed significant improvement in cognitive tests as compared to the placebo group [42]. Table 1 summarizes human trials for AD.

**Table 1:** Summary of human trials of stem cell therapy for Alzheimer’s disease.

Author (Year)	Disease	No. of participants	Type of stem cells	Mode of delivery	Patient outcomes
Kim et al (2021) [40].	Alzheimer’s disease.	9	human umbilical cord blood-derived mesenchymal stem cells (hUCB-MSCs)	Injections to right lateral ventricles via Ommaya reservoir	Therapy was feasible, relatively and sufficiently safe, and well tolerated.
Kim et al. (2015) [39].	Alzheimer’s Disease.	9	Umbilical cord derived stem cell.	Stereotactic injection	No adverse reactions reported other than headache and wound pain.
Rash et al. (2025) [41].	Alzheimer’s disease.	120	Bone marrow derived stem cells.	Intravenous infusions.	Improvement in cognitive tests among patients. Reduction in neuroinflammation.
Brody et al. (2023) [42].	Alzheimer’s disease.	33	Allogeneic mesenchymal stem cells.	Intravenous infusion.	Subjects in low dose group demonstrated improvement in cognitive tests.

Huntington’s Disease

Huntington’s disease (HD) currently lacks a sufficient pharmacotherapy. Antipsychotic and antiepileptic medications are considered the only palliative therapies for these symptoms. Mouse trials have shown promising results of SCT. A prime example is the YAC128 mouse model trial in which bone marrow MSCs were utilized and have shown motor improvement and neuronal loss reduction [43]. Similarly, the R6/2 mouse model demonstrated MSCs' therapeutic effects on the loss of medium spiny neurons, striatal atrophy, HTT aggregation, and stimulation of endogenous neurogenesis [44]. The YAC128 mouse model used induced pluripotent stem cell-derived neural stem cells and therapeutic effects on neuronal loss and increased BDNF and tropomyosin receptor kinase (BTrkB) levels in the striatum [45].

Currently, there is only one clinical trial underway [NCT032535, NCT02728115, NCT04219241]. iPSCs derived from fetal or adult brain neural progenitor cells (NSCs) have a staggering potential for neurodegenerative diseases, such as HD. So far, the NSCs have been tested on HD animal models by their direct transplantation into the striatum. Positive results have been illustrated on both motor and psychological symptoms [46]. A common challenge of the majority of approaches that have used NSC seems to be their limited ability to differentiate fully functional, pre-synaptic, or post-synaptic competent neurons. New methods must be established for the in vitro differentiation of functional, reliable markers. The transformation of somatic cells into pluripotent stem cells through the reprogramming of specific transcription factors can be utilized for personalized cell therapies. At the current state, the best characteristic HD iPCS lines have been manufactured from the patient fibroblasts using lentivirus or retrovirus for the expression of multiple pluripotency factors, including Oct3/4, Klf-4, Sox2, C-Myc, SSEA3, LIN-28, NANOG, and p53 shRNA [47,48]. Table 2 summarizes animal model trials for HD.

Parkinson's Disease

Parkinson’s disease (PD) is a progressive disease of the nervous system characterized by a decline in the structure and function of dopaminergic neurons in the substantia nigra of the basal ganglia. Current pharmacological and surgical treatments merely improve the symptoms and prove to be less efficacious with the progression of PD, with unfavorable side effects [3,37].

Intranasal transplantation of neural stem cells indicates a novel approach to PD treatment. A single-center, dose-escalation (1.5 million, 5 million, and 15 million stem cells) study with intranasal transplantation of ANGE-S003 human neural stem cells on 18 patients with advanced PD reflected no adverse side effects from ANGE-S003 and safety concerns. 16 patients on follow-up demonstrated improved performance on the Movement Disorder Society-Unified Parkinson's Disease Rating Scale, with peak improvement noted in the 6th month [49]. There has been conflicting evidence on the optimal stem cell type for PD therapy. ESCs and iPSCs have garnered importance due to their convenience in terms of availability and preclinical success [50,51]. A study conducted by Brederlau et al. demonstrated increased chances of teratoma formation in rats grafted with human embryonic stem cells (hESCs) of shorter duration of differentiation in vitro [52]. A preclinical study conducted on monkeys grafted with human-derived iPSCs reflected improved spontaneous movement regardless of whether cells were derived from a healthy person or a PD-afflicted patient. A study showed no tumorous growth for 2 years in cells sorted by a floor plate marker called Corin, serine peptidase (CORIN) [53]. PSCs could potentially be cancerous, but genomic quality control is explorable [54]. A meta-analysis study conducted on animal models of PD treated with adipose-derived stem cells (ADSCs) demonstrated recovery by assessment of the rotation behavior test. The ADSC infusion resulted in long-lasting effects [55]. The discovery of markers to differentiate dopamine-producing progenitors developed from human ESCs led to the development of a good manufacturing practice (GMP) differentiation protocol for efficient production of transplantable dopamine progenitors [56]. A study conducted by Rahimi et al. showed longer survival of bone marrow stem cells due to protection from 1-methyl-4-phenyl-2,3,4,6-tetrahydropyridine among PD rats injected with caffeic acid phenethyl ester [57].

Human fetal ventral mesencephalic tissue (hfVM) transplant among patients with PD has shown variable outcomes. While some patients were weaned off PD medications due to remarkable improvements in rigidity and bradykinesia, other patients did not reveal favorable outcomes. PD-associated pathologies, including Lewy bodies and alpha-synuclein inclusions, have also been observed in long-term grafted dopaminergic neurons. The TRANSEURO open-label transplant study for PD was commenced in 2012 to address this issue, recruiting younger-onset patients with PD (NCT01898390). The trial was due to be completed in 2021, although no update has been provided yet [58]. A recent phase I clinical trial studied the efficacy of intraspinal transplanted hypoxia-preconditioned olfactory mucosa (hOM)-MSCs in patients suffering from severe PD. The SCT modulated the neuroinflammation with no deleterious effects and optimized the recovery of neurological function [59]. A preclinical study used human-induced pluripotent stem cell (hiPSC)-derived midbrain dopaminergic cells (mDACs) from 4 PD patients and injected them into the rodents. mDACs from all cell lines satisfied the safety criteria. However, one rodent did not manifest improved outcomes [60].

Amyotrophic Lateral Sclerosis

Amyotrophic lateral sclerosis (ALS) is a neurodegenerative disease affecting the upper and lower motor neurons, which is characterized by weakness and paralysis. Riluzole, edaravone, and sodium phenylbutyrate/tauroursodiol are the FDA-approved pharmacological treatments to improve quality of life and alleviate symptoms with no cure yet [61]. Berry et al. enrolled 48 ALS patients, among whom 36 participants received MSC-neurotrophic factor (NTF) cells and the remaining 12 received a placebo with 6 months of follow-up. The study exhibited that MSC-NTF was safe and potentially efficacious, with functional improvement among patients with ALS. Cerebrospinal fluid (CSF) neurotrophic factors increased and inflammatory biomarkers decreased in treated participants post-transplantation [62]. A study conducted by Barczewska et al. depicted a two-fold increase in median survival time, with a favorable risk-benefit ratio and no adverse reactions to the treatment of 67 patients with Wharton's jelly-derived MSCs [63]. A study by Siwek et al. showed that repeated intrathecal injection of autologous bone marrow-derived MSCs was safe. ALS patients with a fundamentally rapid course manifested slowing of disease progression. However, no significant changes were observed in patients with slower advancement of the disease. The prolonged preparation time of bone marrow-derived MSCs poses a notable challenge in the clinical setting [64]. Cudkowicz et al. conducted a study administering participants with three doses of autologous MSCs induced to secrete high levels of neurotrophic factors (MSC-NTF) intrathecally. The study did not show a statistically significant outcome due to the small sample size. However, patients in the MSCs treatment arm maintained superior function as compared to the patients receiving placebo [65].

A study conducted in animal models by Baloh et al. suggested that human neural progenitor cells transduced with glial cell line-derived neurotrophic factor (GDNF), developing into astrocytes, are potentially protective of motor neurons in ALS patients and act by blocking dysfunctional astrocytes. No adverse effects were noted one year post-trial. Postmortem reports of 13 participants showed graft survival, GDNF production, and neuroma growth at the injection site [66]. Mazzini et al. enrolled 18 ALS patients in the phase I clinical trial and microinjected them with human NSCs intraspinally. A temporary reduction in disease advancement of the ALS Functional Rating Scale Revised (ALSFRS-R) was observed one month post-transplant, lasting up to four months. No serious adverse effects were noted up to 60 months post-procedure [67]. A clinical trial with 21 ALS patients received three autologous bone marrow-derived mesenchymal stem cells concurrently intrathecally and intravenously with a one-month interval. Steadiness in ALSFRS and forced vital capacity (FVC) values was noted. Serum and CSF evaluation of microRNAs, miR-206, 133a-3p, and 338-3p proved insignificant [68]. Patient-derived iPSCs are used to explore the potential of pharmacological drugs. Ropinirole, retigabine, and bosutinib are among the few pharmacological drugs being administered in human trials to prove efficacy that have been identified using the iPSC-based drug discovery method [69,70]. Table 2 summarizes the human trials for PD and ALS.

**Table 2:** Summary of human trials of stem cell therapy for Parkinson’s disease and amyotrophic lateral sclerosis. PD: Parkinson's disease, ALS: amyotrophic lateral sclerosis, ALSFRS: amyotrophic lateral sclerosis functional rating scale, FVC: forced vital capacity.

Author (Year)	Disease	No. of participants	Type of stem cells	Mode of delivery	Patient outcomes
Jiang S et al. (2018) [49].	PD	18	ANGE-S003 human neural stem cells	Intranasal transplantation	Improved performance on MDS-UPDRS with peak at 6^th^ month with no serious adverse effects
Barker et al. (2019) [58].	PD	11	Neural Allo-Transplantation with Fetal Ventral Mesencephalic Tissue	Surgical injection (transplantation).	Grafted cells can survive over 10 years, reduced bradykinesia and rigidity. Sustained re-activation of motor cortical areas.
Berry et al. (2019) [62].	ALS	36	mesenchymal stem cell (MSC)-neurotrophic factor (NTF) cells	combined intrathecal and intramuscular administration	Safe and early promising signs of efficiency.
Barczewska Monika et al. (2020) [63].	ALS	67	Wharton's jelly mesenchymal stem cells	Intrathecal administration	The stem cells proved safe and efficacious. Female participants responded better.
Siwek et al. (2020) [64].	ALS	8	Autologous bone marrow-derived mesenchymal stem cells	Intrathecal administration	Disease progression decreased in patients with rapid course ALS.
Mazzini et al. (2019) [67].	ALS	18	Human neural stem cell	Micro transplantation into spinal cord	Decrease in disease progression with no side effects up to 60 months.
Alkhazaali-Ali et al. (2025) [68].	ALS	21	Autologous bone marrow-derived mesenchymal stem cells	Intrathecally and intravenously concurrently	Steadiness in ALSFRS and FVC values.

SCT in spinal cord injury (SCI)

Spinal cord injury (SCI) is a debilitating condition that damages the spinal cord, leading to motor, sensory, and autonomic dysfunctions. A diverse range of etiologies contributes to spinal cord injury, including vascular, congenital, inflammatory, infectious, and predominantly traumatic causes, which account for approximately 80% of the total cases. Integrating stem cells into the injured areas leads to the formation of new neural connections and helps restore lost functions. Furthermore, they contribute to creating a supportive environment that enhances cellular survival and regeneration, making them essential for neurological recovery and tissue repair [17,71].

The American Spinal Injury Association (ASIA) Impairment Scale is frequently used to measure the neurological level of functional impairment. Grade A is defined as complete loss of function, grade B is defined as complete loss of motor function but preserved sensory function, grades C and D are defined as incomplete loss of motor function, and grade E is defined as normal motor and sensory functions [71,72]. Shang et al. conducted a comprehensive meta-analysis of 62 studies, involving 2,439 patients. Notably, 48.9% of the patients showed at least a grade improvement on the ASIA Impairment Scale. Additionally, SCT led to substantial improvements in urinary and gastrointestinal function, with increases of 42.1% and 52.0%, respectively. However, 28 adverse effects were reported, including neuropathic pain, abnormal sensations, muscle spasms, vomiting, and urinary tract infections, each affecting more than 20% of patients [72]. In another phase I clinical trial, 10 patients intrathecally administered with adipose-derived mesenchymal stem cells (ADMSCs) were observed for two years. The average patient age was 34.6 years. Seven patients showed improvement in the ASIA impairment scale (AIS) grade post-infusion. The study met its primary endpoint, showing that AD-MSC harvesting and administration were well-tolerated in traumatic SCI patients [73].

In a phase I/II clinical trial, 20 subjects with a minimum of one year since their SCI were recruited. Participants in group A were administered perilesional bone marrow mesenchymal stem cells (BMMSCs), followed by three monthly intrathecal BMMSC injections. In contrast, Group B received three monthly intrathecal umbilical cord mesenchymal stem cell (UCMSC) injections. Both groups exhibited substantial enhancements in their total ASIA scores, with Group A showing more pronounced motor improvements [74]. A phase II clinical trial assessed the efficacy of combined intrathecal injection of Schwann cells (SCs) and bone marrow-derived mesenchymal stem cells (BMSCs) among patients suffering from SCI. 37 (55.2%) and 30 (44.8%) patients in the treatment and control groups were tracked for six months. The treatment group showed considerable drops in mean interference item scores (p<0.001). These scores were evaluated on the basis of daily tasks, mood, and sleep. Substantial improvement in the neuropathic pain was reported by the participants in the treatment cohort [75]. A randomized phase II clinical trial involved 32 patients with complete SCI-induced neurogenic bladders. The combined intrathecal administration of BMSCs and SCs significantly improved urodynamic parameters, reduced urinary incontinence rates, and enhanced quality of life [76]. A study enrolled 14 patients with SCI with impairment duration ranging from 3 to 28 months, transplanted with ADMSCs. Six patients suffered from cervical injury, one from cervico-thoracic injury, six from thoracic injury, and one had a lumbar injury. At the eight-month follow-up, five patients demonstrated ASIA motor score improvements of one to two grades across different outcomes. Additionally, two patients regained voluntary anal sphincter contraction. In terms of sensory recovery, 10 patients showed improvement, while one experienced degeneration. Despite these functional gains, MRI scans showed no structural changes between the baseline and eight months post-transplant, highlighting the need for further investigation into the long-term effects of stem cell therapy [77]. Table 3 summarizes trials of stem cell use in SCI.

**Table 3:** Summary of human trials of stem cell therapy in spinal cord injury

Author (Year)	Disease	Type of study	No. of participants	Type of stem cell administered	Mode of delivery	Patient outcomes
Akhlaghpasand et al. (2025) [75,76].	Complete Spinal Cord Injury with neuropathic pain.	Phase II randomized active- controlled trial.	44 patients.	Mesenchymal stem cells (MSCs) and Schwann cells.	Intrathecal injection.	Significant improvement in neurogenic bladder function. Some patients also experienced secondary motor and sensory improvements. No serious adverse events were reported.
Awidi et al. (2024) [74].	Chronic Spinal Cord Injury (SCI)	Phase I/II clinical trial.	14 patients.	Expanded mesenchymal stromal cells (MSCs) from bone marrow and umbilical cord origins	Intrathecal injection.	Umbilical cord-derived MSCs showed slightly better outcomes compared to bone marrow-derived MSCs. No serious adverse events were observed. Some patients experienced improvements in motor function and bladder control.
Bydon et al. (2024) [73].	Traumatic Spinal Cord Injury (SCI).	Phase I clinical trial.	10 patients.	Adipose-derived mesenchymal stem cells (AD-MSCs).	Intrathecal injection	No serious adverse events were reported. Some participants showed improvement in motor and sensory scores, especially those with incomplete injuries. Neuropathic pain and quality of life measures also improved in a subset of patients.
Shang et al. (2022) [72].	Spinal Cord Injury (SCI)	Systemic review	2,399 patients across 62 clinical trials.	Mesenchymal stem cells (MSCs), and neural stem/progenitor cells (NSPCs).	Intrathecal injection, intraspinal injection, intravenous injection, and surgical implantation	43.2% of patients showed at least one-grade improvement on the ASIA scale. Sensory function showed modest improvement. Mild to moderate adverse events were common; serious adverse events were rare.
Hur et al. (2016) [77].	Spinal Cord Injury (SCI)		14 patients.	Autologous adipose-derived mesenchymal stem cells (ADMSCs).	Intrathecal administration via lumbar tapping.	Motor and sensory function improved in patients with incomplete spinal cord injury. No severe adverse events were reported. Improvements were observed in neuropathic pain and bladder function in some cases.

Limitations and challenges of SCT

While stem cell therapies show promise, it is debatable if they are a definitive cure for neurodegenerative diseases and SCI. Recovery outcomes vary widely among individuals, making it difficult to predict their effectiveness for every patient. One major challenge is the complex structure of the spinal cord and the nature of the injury itself. Simply replacing the lost cells is insufficient. Restoring neural connections and functional pathways remains a substantial challenge. Additionally, the timing of stem cell administration plays a crucial role in recovery. Delayed treatment can reduce the effectiveness of the SCT, as the disease may have progressed considerably. Stem cell survival and integration are a crucial obstacle. After transplantation, factors like inflammation and immune rejection can impair their ability to function properly [17,78]. To improve outcomes, combining stem cell therapy with other treatments such as rehabilitation, pharmacological agents, or surgical interventions may be necessary. This underscores the complexity of neurodegenerative diseases and SCI recovery and the need for continued research to refine treatment strategies [78,79].

There are different delivery methods for stem cell therapy. The two main categories are invasive and noninvasive. The mode of administration of the stem cells also plays an important role in the outcomes. Intravenous (IV) routes' primary limitations include the inability of stem cells to efficiently cross the blood-brain barrier and the risk of entrapment in the pulmonary system, which reduces their availability for spinal cord repair [80]. There is a need to develop standardized protocols for intrathecal delivery regarding dosage, frequency, and administration intervals [71,77]. Intraspinal injection requires a precise evaluation of the injured region for safe and effective infusions. It is crucial to image the epicenter using real-time ultrasound (US) prior to initiating the injection to accurately identify the injury site and determine the optimal tissue pathway for entry. Visible swelling of the spinal cord during the injection process signifies a high risk of pressure injury [81]. Scaffold-based delivery is a viable option. However, to enhance outcomes, it is imperative to precisely formulate a refined scaffold composition to provide sufficient neurotrophic factors [82]. The intranasal route of administration has shown the most potential. It allows rapid absorption and direct access to the central nervous system (CNS). The nasal cavity’s unique anatomical connections bypass the blood-brain barrier, reaching the spinal cord more efficiently. However, further studies are warranted to determine its optimal clinical use [83].

A common challenge faced is the limited ability of NSCs to fully differentiate into functional, pre-synaptic, or post-synaptic neurons. New methods must be established for the in vitro differentiation of functional neural MSCs, expressing reliable markers. To unlock the full potential of the SCT, the desirable cell type must be successfully differentiated, which unfortunately has not been achieved yet due to the lack of the sole generation of homogeneous cell lines. Thus, the risk of cancer due to residual undifferentiated cells is a significant concern. The amount of stem cells retrieved from a person is limited, plus the collecting process can be intrusive and dangerous. Less invasive stem cell implantations are currently being explored in order to minimize the impact. The possibility of immune rejection and tumor formation is an alarming safety issues that need to be addressed [84]. One of the most significant challenges in advancing stem cell therapies for neurodegenerative disorders and spinal cord injuries is scaling up from preclinical to clinical settings. While animal models have provided valuable insights, the complexity of human diseases often exceeds that of preclinical models, necessitating rigorous validation before clinical translation. Large-scale production of stem cells and neurotrophic factors must meet stringent regulatory standards, including GMP guidelines, to ensure safety and efficacy. Additionally, the high costs associated with scaling up, including cell culture, quality control, and delivery systems, pose financial barriers that must be addressed to make these therapies accessible. Another critical challenge is ensuring reproducibility and standardization across different studies and clinical trials. Differences in the genetic background of iPSCs or the composition of biomaterials used for delivery can significantly impact therapeutic efficacy [82-84].

Ethical and regulatory concerns in SCT

Ensuring patients understand the potential benefits and risks of experimental stem cell treatments, which are inherently risky, is crucial for ethical standards and patient autonomy. Informed consent and protecting patient rights are vital in stem cell therapy, especially for neurodegenerative diseases. Patients should completely understand the risks and benefits, including uncertainties like tumor formation or immune rejection [85]. Transparency and trust are key, especially with embryo-derived stem cells. hESCs raise ethical concerns due to the potential harm to human embryos during collection. This necessitates an ethical analysis of hESC applications in regenerative medicine. Critics argue that embryo destruction infringes on their potential for life. This controversy has led to interest in alternative stem cell sources like iPSCs, considered more ethically permissible. Patients can refuse or withdraw without losing other treatment options. Informed consent is ongoing, and patients should be updated on newly emerging risks or developments [85,86]. 

Established in 2002, the International Society for Stem Cell Research (ISSCR) became the leading global science-based organization dedicated to stem cell research and its clinical translation. In addition to fostering scientific discourse and data sharing, the Society developed guidelines promoting high standards in practical and ethical research and its applications [87]. The Beijing Declaration (2023 Xi’an Version) by the International Association of Neurorestoratology (IANR) updates international standards for neurorestorative therapies, promoting ethical practices, global collaboration, and standardized clinical protocols. It addresses the gaps in earlier guidelines and reflects the latest advances in stem cell applications for neurological diseases [88].

A critical challenge arises from the absence of standardized international regulations, leading to inconsistent approval procedures and “stem cell tourism” to nations with lax laws [86,89]. Additionally, the extensive safety and efficacy testing mandated by regulatory entities such as the FDA and European Medicines Agency (EMA) hinders the timely availability of therapies, particularly for complex neurological disorders with uncertain long-term outcomes. Intellectual property issues complicate the progress, as patents restrict access and acquisition costs increase. Personalized stem cell therapies further complicate regulatory frameworks, requiring tailored evaluation and approval. Equity and accessibility remain major concerns. If stem cell therapies become commercially available, cost and availability may create disparities in access, limiting treatment options for certain populations [85,86]. In general, these regulatory obstacles impede stem cell treatments for neurological disorders, necessitating international cooperation and revised frameworks to guarantee safe and equitable access.

Future perspectives

Various gene modification techniques are employed with various delivery vectors to modify underlying genetic contributors to neurologic disorders. Genetically modified NSCs have shown not only good survival but also enhanced resilience in adverse environments such as strokes, SCI, traumatic brain injuries, and neurodegenerative insults. It has been demonstrated that genetic modifications, such as the overexpression of neurotrophic factors BDNF and GDNF, improve cellular differentiation into neurons and glial cells, which in turn significantly contributes to cell survival [90]. The integration of biomaterials with stem cells has emerged as a transformative approach for treating neurodegenerative diseases and spinal cord injuries. Biomaterials such as hydrogels, electrospun nanofibers, and 3D-printed scaffolds provide a supportive microenvironment that enhances stem cell survival, differentiation, and integration into damaged tissues. Different biomaterials are used to achieve these scaffolds, including natural, synthetic, and biodegradable polymers. Injectable hydrogels offer minimally invasive delivery of stem cells, making them particularly suitable for treating conditions such as ALS [91]. Personalized medicine has revolutionized regenerative therapies by tailoring stem cell-based treatments to individual patients’ genetic, molecular, and clinical profiles. Clustered regularly interspaced short palindromic repeats (CRISPR-Cas9) gene editing further enhances this approach by correcting genetic mutations or engineering stem cells to overexpress therapeutic factors, as demonstrated in clinical trials for sickle cell anemia and beta-thalassemia. Biomaterials, such as 3D-printed scaffolds and hydrogels, are customized to support stem cell survival and integration, particularly in tissue engineering for spinal cord and cartilage repair [92].

## Conclusions

Recent stem cell therapy advances show promise in treating neurodegenerative disorders and spinal cord injuries. These therapies may revolutionize medicine, offering innovative treatment options that could replace conventional therapies to attain complete recovery. However, the majority of the potential SCT avenues are in the preclinical stage and require large human trials for approval. Also, there is a dire need to address ethical and legal considerations that must be carefully considered to ensure responsible and equitable use.
